# Adipose tissue and sustainable development: a connection that needs protection

**DOI:** 10.3389/fphar.2015.00110

**Published:** 2015-05-27

**Authors:** Angelo Tremblay, Éliane Picard-Deland, Shirin Panahi, André Marette

**Affiliations:** ^1^Department of Kinesiology, Laval UniversityQuebec City, QC, Canada; ^2^Centre de Recherche de l’Institut Universitaire de Cardiologie et de Pneumologie de QuébecQuebec City, QC, Canada

**Keywords:** energy balance, obesity, sleep, pollution, mental work

## Abstract

Obesity is generally considered as an excess body fat that increases the risk to develop ergonomic, metabolic, and psychosocial problems. As suggested in this paper, body fat gain is also a protective adaptation that prevents body lipotoxicity, contributes to the secretion of molecules involved in metabolic regulation, and dilutes lipid soluble persistent organic pollutants. Recent literature shows that this protective role of adipose tissue is more solicited in a modern context in which unsuspected factors can affect energy balance to a much greater extent than what is generally perceived by health care professionals. These factors include short sleep duration, demanding mental work, and chemical pollution whose impact is more detectable in a context dominated by economic productivity and competitiveness. Since these factors might also include the increase in atmospheric CO_2_, it is likely that obesity prevention will need the support of a promotion in sustainable development, whether it is for human health, and well-being or global ecological protection.

Obesity involves a wide and interactive range of genetic, biological, behavioral, and societal factors contributing to variations in its prevalence. The first law of thermodynamics provides a simple definition of obesity which is the result of an excess in energy intake over expenditure leading to an increase in stored energy as body fat. This increase in body fatness is associated with numerous adverse effects which impact body functionality and the ability to accomplish daily activities. This inconvenience includes ergonomic problems which affect gait, posture and displacement and increase the risk of falls and injuries. The obese individual is also frequently less metabolically healthy with the consequence and likelihood of developing diseases such as diabetes, CVD, and sleep apnea. Finally, excess body fat contributes to suboptimal feeding behaviors and sociopsychological traits that exert a negative influence on health and well-being.

Traditionally, the treatment of obesity and related complications has strictly focused on the first law of thermodynamics since low and very low calorie diets that were commonly prescribed were based on simple diet adjustments to achieve a negative energy balance. This restrictive approach was not inappropriate but failed to consider the body’s biology underlying the matching between energy intake and expenditure. In practical terms, the inevitable outcome has been body weight regain in a context where regulatory processes could not sufficiently adapt to the long-term consequences of caloric restriction.

Today, excess body fat is considered more as a complex problem reflecting the difficulty of the body to be in harmony with its environment. This suboptimal body-environment interaction is partly attributable to unsuspected factors linked to the present socioeconomic context that solicits body fat gain as a protective adaptation.

The main function of adipose tissue is probably to store lipids along with its remarkable endocrine role as revealed by the ever increasing number of immuno-metabolic factors that are found to be released by fat cells (Bluher and Mantzoros, 2015). Lipid storage can accommodate a quasi unlimited positive lipid balance due to the potential hypertrophy and hyperplasia of fat cells. In the obese individuals supervised in our previous weight-reducing interventions, the theoretical excess body fat is about 20 kg in both men and women, as shown in Supplementary Table S1. In addition, the estimates presented in this table suggest that these obese individuals should lose about half of their body fat to reach the theoretical threshold of a healthy body weight.

The clinical experience of our research team includes repeated attempts to promote a body weight loss in obese individuals that could “normalize” body weight and fat. In some of our studies, food habits and physical activity practices were modified and monitored to favor a negative energy balance to the point of resistance to further lose body fat (Tremblay et al., 1991; Doucet et al., 1999; Chaput et al., 2005). Although the outcomes were beneficial, we have never been able to induce morphological changes approaching the baseline theoretical target. Interestingly, the achievement of a threshold of resistance to further lose fat was accompanied by a significant increase in appetite sensations (Doucet et al., 2000a), a greater than predicted decrease in energy expenditure and leptinemia (Doucet et al., 2000b; Tremblay and Chaput, 2009), and a more pronounced trend toward hypoglycemia (Tremblay et al., 1999). These changes are concordant with the results obtained by other investigators in the same research context (Leibel et al., 1995; Rosenbaum et al., 2005). Globally, these observations raise the possibility that some protective functions of adipose tissue may underlie the resistance to lose fat to “normality” in individuals prone to obesity.

## Is Obesity Adequately Perceived?

As indicated above, the main role that has been traditionally attributed to adipose tissue is the storage of excess energy/lipid. This effect is important since it protects non-adipose organs from the lipotoxicity that would necessarily occur when fat intake exceeds fat oxidation. The quantitative aspects of the role of fat storage in the context of overfeeding were evaluated in the Quebec twin overfeeding study in which an excess in energy intake was imposed over 100 days to young adult male twins (Tremblay et al., 1992). As shown in **Table 1**, the energy equivalent of fat gain (210 MJ) corresponded to about 60% of the excess energy intake. This result also emphasized that under conditions of substantial overfeeding, fat storage can explain a greater proportion of the disposal of excess energy/lipid intake compared to thermogenesis.

**Table 1:** Estimated mean energy balance in men subjected to 100 days of overfeeding.

	MJ
Excess energy intake	353
Body energy gain	
• Fat	210
• Fat-free mass	12
Estimated increase in energy expenditure	110
Unexplained energy expenditure	31

*Adapted from Tremblay et al. (1992)*.

The discovery of leptin, Zhang et al. (1994) has confirmed the key role of adipose tissue in some regulatory processes underlying energy metabolism. Although the lipostatic theory of appetite control was proposed as early as the 1950s (Kennedy, 1953), no adipose tissue-related messenger with the potential to transmit a satiating signal had been subsequently identified up until this discovery. Following its identification and initial characterization, leptin was found to promote a reduction in body weight, and food intake (Pelleymounter et al., 1995). It was also found to be increased in obese people (Considine et al., 1996), suggesting a state of leptin resistance in individuals prone to obesity. In response to a weight-reducing program, an early decrease in leptin was observed and found to be related to the greater than predicted decrease in energy expenditure (Doucet et al., 2000b). The definitive proof of involvement of leptin in the metabolic response to weight loss was provided by the group of Leibel and Rosenbaum (Rosenbaum et al., 2005) when they partly restored baseline energy expenditure and sympathetic nervous system activity with the administration of leptin in the weight-reduced obese state.

The leptin discovery has been followed by the identification of other bioactive factors produced by fat cells such as resistin (Steppan et al., 2001) and adiponectin (Yamauchi et al., 2001) which also exert a significant impact on energy metabolism and related variables. Today, the potential of adipose tissue to secrete numerous molecules involved in immuno-metabolic regulation is well recognized. This also highlights the capacity of adipose tissue to contribute to body homeostasis via its secretory potential.

Adipose tissue can also support body homeostasis via the uptake and dilution of lipid soluble compounds classified as persistent organic pollutants (POPs). These compounds are man-made chemical products which include high-performance, low-cost insecticides which are still present in the body of every individual on the planet despite their withdrawal from agricultural use in many countries several decades ago. Their persistence is partly explained by their long half-life and because they can be transported in the air from countries where they are still used toward cold areas of the planet (Ma et al., 2003). Several years ago, Lee et al. (2007) examined the association between five subclasses of POPs and metabolic health and found that organochlorine (OC) pesticides were strongly and consistently related to the development of the metabolic syndrome. Specifically, the odds ratio of displaying the syndrome was more than five times greater in the quartile of more polluted individuals compared to the lower quartile of study participants. As described in the next section of this paper, the management of body levels of POPs may become problematic in the context of a weight-reducing program.

In summary, scientific evidence shows that adipose tissue is not only a body storage compartment of excess energy/lipid intake but is involved in regulatory processes and also attenuates the exposure of target organs to body pollutants. As further discussed, this protective role of adipose tissue seems to be even more essential in a world where economic preoccupations dominate human development.

## About Unsuspected Determinants of Obesity

According to the World Health Organization (2000), obesity has reached an epidemic state which has stimulated numerous public health agencies to develop programs aiming at the promotion of healthy body weight. In general, these programs have been based on healthy eating and an active lifestyle and have targeted people in all age categories. Despite these significant efforts, the prevalence of obesity has not decreased and has even increased (Flegal et al., 2010). Interestingly, this observation echoes the results of Klimentidis et al. (2011) who examined the profile of over 20,000 animals representing eight species living with or around humans in industrialized countries. It was found that average mid-life body weights have risen among primates and rodents living in research colonies, as well as among feral rodents and domestic dogs and cats. On the basis of these results, the investigators suggested “the intriguing possibility that the etiology of increasing body weights may involve several as-of-yet unidentified and/or poorly understood factors” (Klimentidis et al., 2011). This is concordant with our recent research experience demonstrating that non-caloric and non-traditional factors may have a better predictive capacity of the risk of overweight compared to participation in physical activity and diet macronutrient composition (Chaput et al., 2009). In the present paper, we describe the potential impact of some of these factors which are representative of the features of our modern lifestyle.

### Sleep Duration and Quality

As early as the 1990s, epidemiological data documented the existence of a possible relationship between habitual short sleep duration and the proneness to overweight (Locard et al., 1992). Further laboratory standardized testing has confirmed the validity of this association. For instance, an experimentally controlled restricted sleep duration was shown to acutely decrease leptinemia and increase plasma ghrelin and cortisol concentrations and desire to eat (Spiegel et al., 1999, 2004). Further investigations demonstrated that short sleep duration induced a substantial increase in energy intake (Nedeltcheva et al., 2009; Brondel et al., 2010) and a decrease in spontaneous physical activity (Schmid et al., 2009). Accordingly, reduction in sleep was found to interfere with the ability to comply with a negative energy balance (Nedeltcheva et al., 2010).

Our previous population studies and clinical interventions are in agreement with these results. In the *Quebec en Forme* Project (Chaput et al., 2006), we observed that short sleep duration was a much better predictive variable of the risk for overweight compared to physical inactivity. Moreover, as shown in **Table 2**, the Quebec Family Study (QFS) showed that short sleep duration was more associated with the risk of overweight compared to a high-fat diet and lack of participation in vigorous physical activity (Chaput et al., 2009).

**Table 2:** Risk factors for overweight in adulthood: the Quebec Family Study (QFS).

Risk factors	Adjusted OR (Cross-sectional)	Additional weight gain vs. a reference group (kg) (6 years follow-up)
Short sleep duration	3.81^∗^	1.65
High disinhibition eating behavior	3.8^∗^	1.46
Non-participation in high-intensity physical exercise	2.03^∗^	1.23
High dietary lipid intake	1.64^∗∗^	0.61
High alcohol intake	1.37^∗∗^	0.39

*^∗^*p* < 0.01, ^∗∗^*p* < 0.05*.

*Adapted from Chaput et al. (2007b)*.

The QFS also showed that short sleepers who become good sleepers attenuate their increase in body fat over time (Chaput et al., 2012). From a mechanistic standpoint, this study confirmed that short sleep duration is related to reduced leptinemia (Chaput et al., 2007b) and to a greater susceptibility to hypoglycemia (Chaput et al., 2007a). Finally, our clinical experience confirmed the findings of Nedeltcheva et al. (2010) regarding the reduced ability of short sleepers to benefit from a weight-reducing program (Chaput and Tremblay, 2012). The latter study also demonstrated that a decrease in habitual sleep quality is related to a reduced response of body weight and fat to a weight loss program.

The relevance of these observations to the current socioeconomic context is certainly reinforced by the statistics of the US National Sleep Foundation that reports a decrease of 1.5–2.0 h of sleep since 1960 (Leproult and Van Cauter, 2010). This decrease in sleep time is not clearly understood but partly explained by our 24 h turbulent environment imposing awakeness and vigilance whether it is for social relationships or labor requirements. This reality also favors the emergence of a dilemma that is well exemplified by the control of plasma leptin levels. On one hand, there is the social pressure promoting short sleep duration which leads to reduced leptinemia. On the other hand, there are the body’s regulatory processes that include body fat gain and its related increase in plasma leptin levels to permit body homeostasis.

### Demanding Mental Work

Industrialization and computerization have promoted a progressive change in the nature of labor in most sectors of economic activity. Specifically, this shift has partly replaced somatic effort relying on muscle by mental work that depends on neuronal activity. From a metabolic standpoint, neurons have less metabolic flexibility than myocytes since they essentially rely on glucose to support their work. This has incited us to examine the impact of mental work on glycemic stability. Our preliminary study (Tremblay and Therrien, 2006) as well as a subsequent investigation (Chaput et al., 2008) demonstrated that demanding mental work accentuates glycemia instability, including the proneness to mild hypoglycemia known to trigger episodes of food intake (Mayer, 1953; Louis-Sylvestre and Le Magnen, 1980).

The study of the acute effects of demanding mental work confirmed the potential of cognitive effort to promote hyperphagia. In a study first conducted at Laval University in female students (Chaput and Tremblay, 2007), a computerized reading-writing task was found to increase *ad libitum* energy intake by 229 kcal during a buffet-type meal served immediately after the task. Interestingly, the mental work session did not induce quantitatively important changes in energy expenditure compared to a relaxing task control. Furthermore, the increase in energy intake happened without a significant increase in feelings of hunger, suggesting that demanding mental work may impair the satiation process leading to the interruption of feeding.

A second study also performed at Laval University in female students showed that computerized tasks increased subsequent energy intake by 200–250 kcal (Chaput et al., 2008). The results of this study also revealed that the increase in glycemia instability, cortisol, and subsequent energy intake was more pronounced in women for whom the mental task was more demanding (Chaput and Tremblay, 2009a,b). This is in agreement with the recent demonstration that performing homework, perceived as stressful by male students is associated with an increase in total and abdominal fat (Michaud et al., 2015).

Chaput et al. (2011) extended the investigation of the effects of mental work to the study of video game sessions. As expected, energy expenditure was slightly increased by video game playing. However, this increase was much lower than the global impact of the session on subsequent energy intake resulting in a substantial positive energy balance.

As for sleep habits, the increase in school-related activities is a strong societal trend compared to sports and television viewing, as documented by Sturm (2005). Children are more and more fueled by the proliferation of new technologies (such as portable internet small devices) and they are now consuming media for the amount of time that most adults spend at work. It has been shown that the presence of a small screen in the sleep environment and screen time were associated with perceived insufficient rest or sleep (Falbe et al., 2015). The contribution of sleep to screen time’s impact on obesity should be considered in future studies. Since performing exercise between a mental work session and a meal prevents positive energy balance (Lemay et al., 2014), it seems relevant to increase participation in physical activity to better balance mental and somatic stimulations whether at school or in work facilities. Although this scenario seems reasonable and justified, it is certainly relevant to emphasize that its potential implementation does not benefit from solid traditions in the industrialized world. In this regard, one of the most relevant examples is the habitual template of items in the negotiation of a working contract between directors of companies and unions of workers that very rarely consider this issue as a priority.

### Chemical Pollution

The case of the disposal of lipid soluble OC compounds represents one of the key examples of the conflict that may oppose economic development and the promotion of human health and well-being. As indicated above, this becomes particularly obvious in the context of a weight-reducing program which decreases the dilution space of the pollutants. In obese individuals who are known to display a greater OC body load than lean controls (Pelletier et al., 2002a), a substantial fat loss has been repeatedly shown to increase plasma (Backman and Kolmodin-Hedman, 1978; Walford et al., 1999; Chevrier et al., 2000), and adipose tissue OC levels (Chevrier et al., 2000). Since this hyperconcentration occurs in a uniform way in every lipid compartment of the body (Pelletier et al., 2003), body fat loss increases the exposure of every target organ to the detrimental effects of these pollutants as a result. In the skeletal muscle, the fat loss-induced changes in some circulating pollutants were related to a greater decrease in skeletal oxidative enzymes than that predicted by body weight loss (Imbeault et al., 2002). Accordingly, we also observed that weight loss promoted a greater than predicted decrease in plasma thyroid hormone (T3) concentrations and resting metabolic rate in obese individuals (Pelletier et al., 2002b). Furthermore, when compared to plasma leptin as a predictor of weight loss-induced changes in sleep metabolic rate, changes in plasma OCs were more associated with those in energy expenditure (Tremblay et al., 2004). In summary, fat loss is associated with metabolic changes that negatively alter the control of energy expenditure in association with the enhancing effect on blood and tissue levels of lipid soluble POPs.

These results also raise the question of the possibility of taking advantage of a fat loss program to develop strategies aimed at the elimination of body OCs. This has been tested by Geusau et al. (1999) who strongly increased the clearance of tetrachlorodibenzodioxin (TCDD) with a supplementation of Olestra chips for 38 days in two individuals highly contaminated by 2, 3, 7, 8-TCDD. This is concordant with data reported by Moser and McLachlan (1999) who found an increase in the excretion of OCs induced by Olestra. In a severely contaminated individual, Redgrave et al. (2005) also observed a considerable decrease in OC concentrations after Olestra supplementation. In a more recent study, we found that the partial replacement of dietary lipids by Olestra in obese individuals subjected to a low-fat diet resulted in a decrease in plasma βHCH which was in contrast to the increase induced by the standard low-fat diet. However, we were unable to detect the same benefit for 18 other compounds of the OC category. From a mechanistic standpoint, Geusau et al. (1999) proposed that the non-absorbable nature of Olestra and its structure analog to lipids accentuates the OC gradient toward the gut and a resulting increase in fecal loss.

The OC profile of sea lions also provides useful information about the ability of adipose tissue to detoxify the body. For instance, Ylitalo et al. (2005) reported a negative relationship between blubber thickness and polychlorinated biphenyls (PCBs). This study also revealed that a low level of blubber fat is related to increased likelihood of carcinomas. The latter observation may also highlight that the ability of adipose tissue to dilute POPs and to protect organs playing a key role in the body’s viability is not unlimited.

## Adipose Tissue and Sustainable Development

Chemical pollution is not the only environmental factor that may solicit the homeostatic protection of adipose tissue. Recent evidence suggests that atmospheric CO_2_ can increase energy intake and thus promote a positive energy balance. According to Hersoug et al. (2012), an increase in atmospheric CO_2_ slightly decreases the pH of body fluids which may be related to an increase in energy intake. These investigators also reported preliminary data reinforcing the idea that a small increase in atmospheric CO_2_ in humans favors a positive energy balance. This is in agreement with evidence documenting a link between CO_2_ and the control of hypothalamic orexin neurons (Williams et al., 2007). These observations obviously deserve confirmation which may reinforce the idea that the management of sustainable development should consider body homeostasis, particularly the solicitation of the protective role of adipose tissue.

## Conclusion

The management of obesity is generally performed via the promotion of healthy eating and an active lifestyle together with adequate psychosocial support. Although such a paradigm is sound and justified, we argue in this paper that the prevention of excess body fat also deserves the promotion of sustainable development. This vision is based on the consideration of the protective role of adipose tissue on body homeostasis which, beyond excess lipid storage, secretes molecules that are essential to some regulatory processes. In addition, adipose tissue plays a key role in body detoxification mostly via the dilution of lipid soluble POPs. In this context, the main idea for obesity prevention has some resemblance with that underlying the promotion of a healthy planet (i.e., not to oversolicit the protective resources of the host). As explained in this paper, this is difficult to manage in a modern world where daily socioeconomic requirements impose to the body (adipose tissue) some non-traditional demands that likely exert a strong adipogenic impact. Furthermore, by considering the rise in atmospheric CO_2_ as a potentially obesogenic factor, it is likely that the foreseeable future might be worse than what we currently experience in terms of the obesity epidemic. In fact, it appears that no significant progress regarding obesity management and sustainable development will be achieved as long as money-making preoccupations will continue to dominate those related to human development. More research, and particularly multifactorial research, is needed to investigate the environmental and socioeconomic determinants of obesity. Experimental studies are also necessary to better understand the impact of environmental determinants such as pollutants on the regulation of energy metabolism. Globally, this research should permit a better understanding of the interaction between biological and lifestyle factors and their impact on the proneness to obesity.

## Conflict of interest statement

The authors declare that the research was conducted in the absence of any commercial or financial relationships that could be construed as a potential conflict of interest.
