# Supplementary material for: Adipose tissue and sustainable development: a connection that needs protection
Source: Front Pharmacol. 2015 May 27;6:110. doi: 10.3389/fphar.2015.00110 (PMC4445306; doi:10.3389/fphar.2015.00110)
Supplement: Supplementary file 1 [file Table_1.PDF]

Supplementary Material

Adipose tissue and sustainable development: a connection that needs protection

Angelo Tremblay*[1,2] ,Éliane Picard-Deland[2],Shirin Panahi[1],André Marette[2]

[1] Department of Kinesiology, Laval University, Quebec, Canada; [2] Centre de recherche de l'Institut universitaire de cardiologie et de pneumologie de Québec

* **Correspondence:** Angelo Tremblay, Department of Kinesiology, PEPS, Room 0234,Laval University, Quebec City,Canada,G1V 0A6,Tel: 418-656-7294,E-mail: angelo.tremblay@kin.ulaval.ca

Supplementary Table. The profile of an obese individual.

**Variables**	**Man**	**Woman**
BMI (kg/m^2)	33.4	34.9
Body weight (BW)*	100.9	89.0
Fat mass (FM)*	37.4	43.0
Fat-free mass (FMM)*	63.5	46.1
BW at BMI 25*	75.5	63.7
FM at BMI 25*†	18.4	24.0
FFM at BMI 25*†	57.1	39.7

* Values are in kg

† Assuming that the composition of Δ BW is 75% FM and 25% FFM

Adapted from Doucet et al. (Doucet, Imbeault et al. 1999)
